# Remote Monitoring of COVID-19 Patients Using Multisensor Body Area Network Innovative System

**DOI:** 10.1155/2022/9879259

**Published:** 2022-09-15

**Authors:** Israa Al-Barazanchi, Wahidah Hashim, Ammar Ahmed Alkahtani, Haider Rasheed Abdulshaheed, Hassan Muwafaq Gheni, Aparna Murthy, Elika daghighi, Shihab A. Shawkat, Zahraa A. Jaaz

**Affiliations:** ^1^College of Computing and Informatics, Universiti Tenaga Nasional (UNITEN), Kajang, Malaysia; ^2^Computer Engineering Techniques Department, Baghdad College of Economic Sciences University, Baghdad, Iraq; ^3^Institute of Sustainable Energy, Universiti Tenaga Nasional (UNITEN), Kajang 43000, Selangor, Malaysia; ^4^Department of Medical Instrumentation Technical Engineering, Medical Technical College, Al-Farahidi University, Baghdad, Iraq; ^5^Computer Techniques Engineering Department, Al-Mustaqbal University College, Hillah 51001, Iraq; ^6^Professional Engineers in Ontario, North York, Toronto, Ontario M2N 6K9, Canada; ^7^Technical and Vocational University, Tehran, Iran; ^8^University of Samarra, Samarra, Iraq; ^9^Computer Department, College of Science, Al-Nahrain University, Jadriya, Baghdad, Iraq

## Abstract

As of late 2019, the COVID19 pandemic has been causing huge concern around the world. Such a pandemic posed serious threats to public safety, the well-being of healthcare workers, and the overall health of the population. If automation can be implemented in healthcare systems, patients could be better cared for and health industries could be less burdened. To combat such challenges, e-health requires apps and intelligent systems. Using WBAN sensors and networks, a doctor or medical professional can advise patients on the best course of action. Patients' fitness could be assessed using WBAN sensors without interfering with their daily activities. When designing a monitoring system, system performance reliability for competent healthcare is critical. Existing research has failed to create a large device capable of handling a large network or to improve WBAN topologies for fast transmitting and receiving patient data. As a result, in this research, we create a multisensor WBAN (MSWBAN) intelligent system for transmitting and receiving critical patient data. To gather information from all cluster nodes and send it to multisensor WBAN, a novel additive distance-threshold routing protocol (ADTRP) is proposed. In small networks where data are managed by the transmitting node and the best data route is determined, this protocol has less redundancy. An edge-cutting-based routing optimization (ES-EC-R ES-EC-RO) is used to find the best route. The Trouped blowfish MD5 (TB-MD5) algorithm is used to encrypt and decrypt data, and the encrypted data are stored in a cloud database for security. The performance metrics of our proposed model are compared to current techniques for the best results. End-to-end latency is 63 ms, packet delivery is 95%, security is 95.7%, and throughput is 9120 bps, according to the results. The purpose of this article is to encourage engineers and front-line workers to develop digital health systems for tracking and controlling virus outbreaks.

## 1. Introduction

The ongoing COVID-19 endemic, poor lifestyle choices, insufficient stress relief, rising healthcare costs, and an aging population all posed significant challenges to governments and healthcare organizations around the world. The growing number of patients has necessitated the use of advanced technologies to enable doctors to remotely monitor patients via wireless body area network (WBAN) sensors [1]. However, because WBAN networks involve remote access and sensitive and critical data, they necessitate extremely high levels of security and privacy during storage and processing. As a result, the installed WBAN infrastructure should always include a variety of security elements to ensure data protection, privacy, integrity, and confidentiality. Existing wearable device platforms are constrained by hardware capabilities, parameter estimation techniques, and software design [2].

An outbreak of a virus such as COVID19 can alter the worldwide health and economic landscape. It can result in massive monetary damages both locally and globally. To protect the healthcare systems from collapsing, the world urgently needs to use the Internet of medical things (IoMT) technologies to help monitor patients and save many lives. MSWBAN plays an essential part in IoMT in the healthcare sector, where multiple sensors are used to monitor various medical symptoms of patients [3]. WBAN necessitates the collection of data from sensor nodes in an effective and efficient manner in order to ensure the network's dependability. The cluster head selection scheme is one of the key schemes that contribute to the WBAN network's increased efficiency. From a group of nodes, a few are chosen as cluster heads (CHs). The data are then obtained by the CHs from “contributing nodes,” which are nodes that are linked to one another. The nodes that send their readings to CH are typically located near the CH [4]. To be used in the clustering approach, the data collected by each sensor must be sent to the sink via the cluster head. When it comes to WSN power usage, clustering algorithm-based hierarchy routing protocols elect and rotate CHs at random. Inefficient CH, regardless of network size, may be chosen in certain circumstances, resulting in variable cluster sizes. Clustering algorithm-dependent hierarchical routing protocols employ a single cluster formation parameter and a probabilistic cluster selection approach. As a result, choosing CHs and building clusters is difficult in clustering algorithm-based hierarchy routing protocols [5].

**Figure 1:**
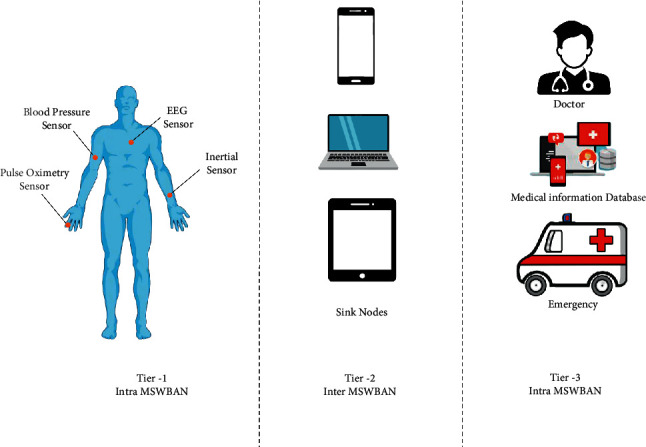
Architecture of MSWBAN.

**Figure 2:**
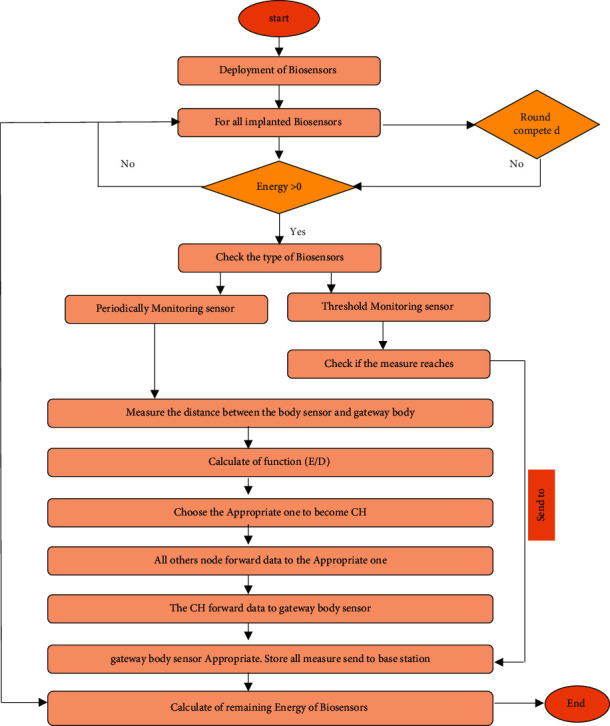
WBAN flowchart.

Figure 1 depicts the MSWBAN's architecture. It has three levels. Wearable or implanted biosensors (ECG, EEG, temperature, blood pressure) capture the data through ZigBee and Bluetooth wireless technologies and transmit it to the body coordinator (BC). Intra-MSWBAN is another name for Tier 1. A BC in Tier 2—also referred to as Inter-MSWBAN—transmits patient data to a nearby access point or sink node. This gateway is a conduit for sharing patient data from Tier-1 to Tier-3. Using the Internet, the sink nodes transfer the gathered data to a distant medical facility or doctor in Tier 3 (also known as Beyond MSWBAN).  Figure 2 shows the WBAN flowchart.

This paper proposes remote monitoring of COVID-19 patients using additive distance threshold routing protocols in the MSWBAN innovative system. The rest of the paper proceeds as follows; Part II contains the relevant literature as well as the problem research gaps. Part III explains the flow of the proposed form. Part IV investigates and compares the behaviour of the proposed method to previously established methods. Finally, Part V concludes the paper.

## 2. Related Works

Singla et al. [6] defined many security criteria for WBANs and conducted a comprehensive evaluation of current secure routing techniques. Many secure routing protocols have been evaluated in terms of security and performance, and an assessment based on these characteristics has been produced, while a comprehensive review of concern about security and privacy is presented about WBAN in [7]. A strategy to bridge the gap between current technological developments and the healthcare system has been presented in [8], in which WBAN sensors and networks may enable a doctor to assist a COVID-19-infected patient in making the best possible decision for themselves at the appropriate time. This situation enables the community to maintain social distance while keeping hospitalized patients in a comfortable atmosphere. Nanosensors are employed in the wireless WBAN to continuously monitor a patient's medical health because of resource constraints and essential applications, improving security and privacy to a high degree offers various challenges. Majumder et al. in [9] conducted a thorough survey in the domain of WBAN. WBAN is a technological breakthrough that has made remote patient monitoring possible. When medical personnel is in low supply, and some patients need 24-hour monitoring, BAN is an important tool for such a scenario.

WBANs are used in both medical and nonmedical applications [10]. It has also thoroughly explored the different wireless technologies that WBAN can support. Routing protocols have affected one of the essential factors of assessing network efficiencies, such as power consumption, throughput, and delay. Researchers may compare routing protocols to help in the development of a certain protocol for a given application. In addition, wireless technologies that employed WBAN systems were investigated in [11]. It comprises miniature sensors that gather and send data across a wireless network, allowing medical professionals to monitor patients in their everyday lives and provide real-time medical diagnoses. Several wireless technologies have already shown value in WBAN applications, while others are still in the research phase. According to Jin et al. in [12], fever, cough, and expectoration were the early symptoms in 36 patients (80%), 23 patients (51%), and 15 patients (33%), respectively, at the commencement of illness. Senior patients (58) and their concomitant chronic diseases were independent predictors of a severe and critically sick population with a higher fatality rate. COVID-19 has the potential to harm a variety of organs in the human body. Treatment of COVID-19 patients with glucocorticoids is well-accepted. Furthermore, Basiri established in [13] that the coronavirus is an encapsulated virus of the RNA virus family. Fever, cough, and shortness of breath are present in the patients, and no definitive therapy or vaccination is available. Due to the body's generation of antibodies, the viral infection progressively becomes self-limiting. Using a novel lab test developed by the Centres for Diseases Control and Prevention, the SARS coronavirus, which causes “severe acute respiratory syndrome,” has been identified. Traditional medicine seems to be beneficial in the treatment COVID-19 sufferers as well.

The major purpose of the framework is to bridge the gap between the current technologies and healthcare systems. WBAN, fog, cloud, and clinical decision support systems are combined to give a comprehensive paradigm for sickness diagnosis and monitoring. The framework is a powerful tool, and they expect it to have a significant impact on the spread of COVID-19 as well as a considerable reduction in healthcare costs [14]. WBSN and contemporary advances in the field were discussed in [15], which emphasized the need for remote monitoring for the elderly and chronically ill. The scientific notion of WBSN architecture, problems, healthcare applications, and their needs was conducted to meet the scientific idea of WBSN. Following that, the key critical part of the WBSN, such as data collection, fusion, risk assessment, and decision-making, is explored. Finally, the article suggests that increasing awareness of critical concerns and the future growth of WBSNs is a great way to monitor a patient's life. According to Qu et al. in [16], the introduction of WBSN has brought hope and a new era in the battle against population aging, chronic illnesses, and a lack of medical facilities. WBANs necessitate the development of routing protocols, which play a crucial role in communication stacks and have a significant impact on network performance. Furthermore, WBSN present issues, applications, and discoveries, and performance difficulties were discussed in [17]. The study covers WBAN Signal processing, network reliability, spectrum management, and security. As a result, they conclude the study by identifying various current and future research directions. On the other hand, a framework for evaluating COVID-19 prevention and protection strategies in hospitals was discussed in [18]. COVID-19 recommendations for preventative and protective measures, tight procedures, frequent audits, staff education and training, and active monitoring should emphasise the case hospital management. During the COVID-19 period, this suggested evaluation model is a novel effort in in-hospital assessment in preventative and safety actions in the healthcare industry. This methodology will serve as a continual evaluation tool for hospital management looking to enhance their COVID-19 prevention efforts. Then, Rahman et al. in [19], demonstrated that WSN frameworks are widely utilised in applications such as healthcare and smart cities to collect and analyse real-time data and take appropriate actions based on demand of the application. Application-specific demands and requirements might play a significant role in deciding on a routing protocol for a WSN. In order to design an inefficient routing protocol, the networking infrastructure must be generalized, while application-specific limitations are ignored. During the quarantine period, a wearable gadget prototype is intended to remotely monitor the COVID-19 health symptoms of potentially infected patients (PIP). The 3D prototype incorporates a three-layer wearable body sensor, a web API layer, and a mobile front-end layer for an automated healthcare system to lessen stress and create a communication channel between physicians, medical authorities, and family responders [20]. WBANs provide information-based sickness diagnosis, allowing for early treatment. If attackers access the web, the whole network will become wasteful. Using biometric and digital signature technologies, this research proposes an integrated security framework that counters intruder assaults and improves network security, resulting in a more trustworthy network and stability [21]. Sangeetha Priya et al. in [22] propose a security-conscious trustworthy cluster established routing protocol for WBS. Many human-centric applications rely on large-scale deployments of wireless body sensor networks. Sensor hubs' vitality reserve funds are among the most crucial components of such systems to extend their life spans. The wireless body area sensor system must also include secure routing to reduce the risk of information leakage. Furthermore, new security concerns has been introduced by WBANs and the services, so that WBAN is evolving to suit these demands [23]. Detection method of COVID-19 is based on a multistage fuzzy rules' technique, with the CART algorithm used to generate the fuzzy rules [24]. The suggested strategy distinguished the growth of illness prediction accuracy according to the implementation outcomes. This study provides detailed specifications of an IoT-based [25] conceptual design for a COVID-19 patient monitoring system. The solution contains method for modifying this assessment approach, as well as ensuring the individualization of evaluations, and a legitimate and widely utilised early-warning score system for evaluating and monitoring hospitalized patients.  Table 1 shows the related works.

## 3. Proposed Work

**Figure 3:**
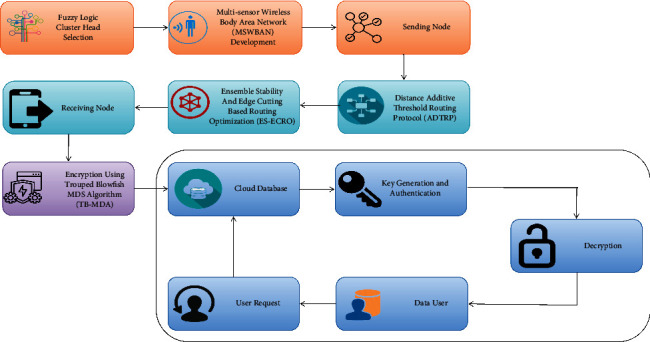
Schematic representation of the suggested methodology.

Figure 3 depicts a schematic illustration of the proposed approach. It includes the process of analysing the fuzzy logic dependent cluster head selection, multisensor wireless body area network deployment, sending node, receiving node, encryption using the trouped blowfish MD5 algorithm, cloud database, key generation, and authentication, decryption, distant monitoring of COVID-19 patients using additive distance-threshold routing protocol in MSWBAN innovative system.

### 3.1. Data User

This component collects personal information as well as serves illness symptoms. There are 493 COVID-infected and 206 noninfected individuals with symptoms, and the data are used to build a threshold routing protocol model. Following that, each new user inputs their information and signs into the system, and the suggested trouped blowfish MD5 algorithm model is used to safeguard COVID-19 patients' data [26].

### 3.2. Fuzzy Logic-Based Cluster Head Selection

#### 3.2.1. CH Selection

Cluster-based routing is an energy-efficient strategy in which high-energy nodes process and transport data as clusters form. Low-energy nodes are in charge of detecting and transmitting data to the cluster heads. The cluster information routing protocol improves scalability, energy efficiency, and security. The longevity of the network should be maximized.

#### 3.2.2. Cluster-Head Selection Using Fuzzy Logic

The FLCH-based clustering technique uses three input parameters to choose wireless sensor networks CHs [27]. Input variables for the model include remaining energy (RE), nodes centrality (NC), and distance to the base station (DBS). The residual energy of a node must be considered while determining whether it belongs to a CH or not. BS receives the information acquired by CH nodes, compiles it, and then sends it on to other nodes. One-hop adjacent nodes in *R*_*c*_are are called Node Degrees (ND) based on the total number of one-hop neighbours. Neighbour-centricity (NC) describes a node when it is located between two other nodes in a ring. When the NC value is low, a node has a better chance of being selected as a CH.

(1) $$\begin{array}{c} NC=\frac{\sqrt{\sum_{i=1}^{ND}dis_{i}^{2}/ND}}{Ntk-Dimension}\text{.} \end{array}$$

The counting of neighbours in node's transmitting radius *R*_*c*_ is denoted in equation (1). In the *M* × *M* field region, “*M*” is the value *Ntk*—*Dimension*, and the distance between *ith* neighbouring nodes is represented by *dist*_*i*_^2^. The amount of energy consumed in data transmission increases the distance between the transmission and reception nodes. When selecting CH, the remaining energy in the sensors, as well as their maximum and minimum distances from the BS, are taken into consideration

(2) $$\begin{array}{c} Distance\;to\;BS=\frac{d_{i}}{\alpha .Ntk-Dimension}\text{,} \end{array}$$

(3) $$\begin{array}{c} \alpha =\frac{d_{\max}}{Ntk-Dimension}\text{.} \end{array}$$

The distance between nodes i and the BS is denoted by*d*_*i*_ and the maximum length among a network node, and BS is *d*_max_ indicated by max. In contrast, *α* implies the network dimensional constant. When selecting the CHs, the remaining energy of the sensor is taken into account, as well as the sensors' and BS's maximum and minimum distances, the amount of energy used by each cluster of sensors, the quality criteria for collections, the sensor distribution, the group mean distance, and the cluster density. The four overall energy levels we proposed are low, medium, and high. They are the energy's “fuzzy linguistic variables” in their totality.

(4) $$\begin{array}{c} Low\left\{\begin{array}{cc} 1 & Energy\leq 0.25\\ \frac{0.35-Energy}{0.1} & 0.25<Energy\leq 0.35 \end{array}\right.\text{,} \end{array}$$

(5) $$\begin{array}{c} Medium\left\{\begin{array}{cc} \frac{Energy-0.25}{0.25} & 0.25<Energy\leq 0.5\\ \frac{0.6-Energy}{0.1} & 0.5<Energy\leq 0.36 \end{array}\right.\text{,} \end{array}$$

(6) $$\begin{array}{c} High\left\{\begin{array}{cc} \frac{Energy-0.5}{0.3} & 0.5<Energy\leq 0.8\\ \frac{0.89-Energy}{0.09} & 0.8<Energy\leq 0.89 \end{array}\right.\text{,} \end{array}$$

(7) $$\begin{array}{c} Very\;High\left\{\begin{array}{cc} \frac{Energy-0.8}{0.09} & 0.8<Energy\leq 0.89\\ 1 & Energy>0.89 \end{array}\right.\text{.} \end{array}$$

The following are the membership functions for the distance parameters:

(8) $$\begin{array}{c} Near\left\{\begin{array}{cc} 1 & x\leq c_{1}\\ \frac{L-x}{L-c_{1}} & c_{1}<x\leq L \end{array}\right.\text{,} \end{array}$$

(9) $$\begin{array}{c} Average\left\{\begin{array}{cc} \frac{x-c_{1}}{L-c_{1}} & c_{1}<x\leq 1\\ \frac{c_{2}-x}{c_{2}-L} & L<x\leq c_{2} \end{array}\right.\text{,} \end{array}$$

(10) $$\begin{array}{c} Far\left\{\begin{array}{cc} \frac{x-l}{L-c_{1}} & L<x\leq c_{2}\\ 1 & x>c_{2} \end{array}\right.\text{.} \end{array}$$

In the equations above, the BS minimum and maximum sensor distances are *c*_1_ and *c*_2_, respectively, and an average distance to the BS is denoted by *L*, which is calculated as follows,

(11) $$\begin{array}{c} L=\frac{\left(c_{1}+c_{2}\right)}{2}\text{.} \end{array}$$

**Figure 4:**
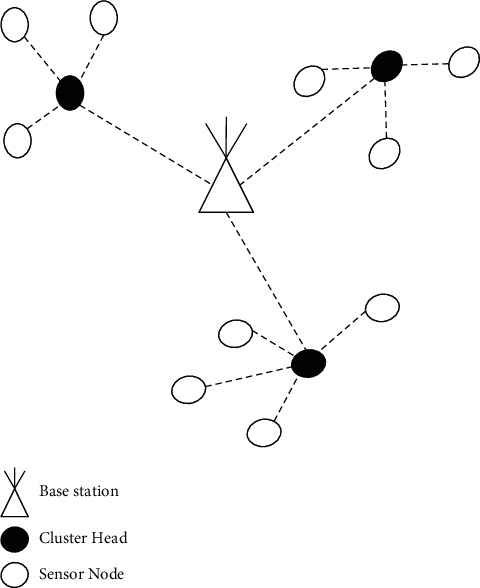
Cluster head.

The CHs are chosen to have the most energy and the shortest distance to the BS. As a result, numerous sensors in each cluster have the potential to be a CH, and the final CH is determined by which sensor best satisfies the set criteria.  Figure 4 depicts the cluster head.

*(1) MSWBAN Development*: the wireless body sensor connects or implants each sensor in multisensor WBAN. These devices monitor electrocardiograms, blood pressure, temperature, heart rate, pulse oximetry, and steps. The MSWBAN architecture may be divided into three subsystems: information capturing, transmitting, and processing. Body sensors oversee collecting physiological data and transferring it to the gateway, which then sends the data to a distant server for analysis. To construct sophisticated MSWBAN systems, the first step is to detect and acquire physiological data about the patient and his surroundings. There has been a rise in the need for a greater depth of information from sensors. Fusing the outputs from many sensors may be the only method to access that breadth of knowledge when a single sensor modality is insufficient. However, in the context of our proposed system, different sensors employ different physical principles, cover distinct information spaces, and provide data in various forms at various sampling rates. The data collected have a various resolution, accuracy, and dependability characteristics. It is essential to utilise a technique that can appropriately fuse data from diverse sources considering these impacts to get the required detection to work successfully. To coordinate a peripheral module and execute the data processing function, data collecting capabilities are equipped with several sensors, wireless data transmitting modules, electricity supply modules, and a microcontroller. MSWBAN's application-based design is inherently static. Infrastructure and application are inextricably linked. The sensors platform, gateway, and server would all have to be updated if the application intelligence were to be changed. Developing a sensor platform, gateway, and remote server from the ground up will need a distinct physical structure for each application. However, deploying a new application is not simple; instead, it involves a lengthy period. Therefore, future application innovations are hampered. Patients using MSWBAN in healthcare typically enjoy complete freedom of movement when body sensors are linked to their bodies. They are occasionally in proximity or within the nearby MSWBAN in such cases. Because of this movement, packets are lost, and the error rate rises. As a result, in MSWBAN, a robust handover mechanism should be provided [27].

*(2) COVID19 Patient Monitoring Via MSWBAN*: MSWBAN is a kind of external monitoring health care system (eHealth), which is a type of continuous health monitoring system that provides local monitoring and control. To treat the ill, these systems do not need frequent hospitalizations. It's a win-win situation since it prevents last-minute scrambling and saves time.

For MSWBAN, most major challenge is its energy consumption since the biosensors it employs have a charging leakage issue and must be replaced after a set of time. An IoT system for the real-time healthcare monitoring systems for the prediction of a preliminary phase of COVID-19 is shown in this study using wearable sensors, including temperature, heartbeat, and pressure sensors. Body temperature, respiratory symptoms, and oxygen levels may all be measured with these biosensors. This gadget communicates biosensor data to the cloud utilizing low-power LoRa technology using Arduino, My Signal hardware, and LoRa technology. To simulate and monitor patients, back-end servers show real-time data, while cloud servers gather, handle, and transport that data [28].

### 3.3. Distance Additive Threshold Routing Protocols

In MSWBAN, there are two types of threshold routing systems: data transmission and data reception. During the threshold routing of aggregated data from CHs to the BS, the routing protocol [6] utilizes an election energy threshold,*T*_*nhCH*_, to choose the next CHs.

(12) $$\begin{array}{c} E_{Uy}\left(k,d\right)=E_{elec}\cdot k+E_{amp}\cdot K.d^{2}\text{,} \end{array}$$

(13) $$\begin{array}{c} E_{sy}\left(k\right)=E_{elec}\times k\text{,} \end{array}$$

where the per-bit dissipation of transmitter circuits is denoted by *E*_*elec*_, the transmitter amplifier dissipation is denoted by *E*_*amp*_, the bit length is given by *k*, whereas the transmission line length among the sender and receiver is given by *d*.

Threshold routing in an MSWBAN is the process of transmitting detected data to the Base station through various protocols rather than sending it directly to the Base station. Every round, threshold routing across CHs involves passing aggregated data via many CHs to the BS. When the CH is next to the BS, information is sent directly to the base station; however, when the distance between BS and CH is significant, high energy is spent in transferring the sensed data to the BS via the radio energy mode. During network configuration, all sensor networks communicate their residual energy (RE) levels and locations to the base station (BS). As a result, the BS has comprehensive awareness of the whole network region. During data routing to the BS, the threshold is utilised to decide which CH should serve as the next protocol.

(14) $$\begin{array}{c} E_{nhCH}=\frac{\sum_{n=1}^{l}RE\left(neighbour\;CHs\right)}{t}, \end{array}$$

where *E*_*nhCH*_ that is, the election energy threshold, the residual energy of neighbour CHs is denoted by RE, and *t* is the number of neighbour CHs.

(15) $$\begin{array}{c} d_{0}=\sqrt{\frac{E_{amp1}}{E_{amp2}}.} \end{array}$$

The threshold value, together with the estimated distances of the nearby CHs, is utilised to determine the following protocol in the threshold route's creation. A distance threshold value, the maximum transmission distance, is determined by *d*_0_.

### 3.4. Encryption Using Trouped Blowfish md5 Algorithm

Blowfish md5 algorithm is a symmetric technique, and the same key is used for encryption and decryption. It is utilised in the encryption process because it is substantially quicker than DES and has a strong encryption rate with no practical cryptanalysis method.

#### 3.4.1. Blowfish Algorithm

Blowfish's cryptographic calculation is productive and customizable, with several parameters (“key size, square size, number of rounds”) that may be utilised to combine certain quality with power consumption and computational overhead. With the right conditions, this blowfish calculation might work well for MSWBAN applications with varying data quantities. The blowfish computation had a positive influence on the cryptography business when compared to algorithms. The author also proposed a multipurpose security system that uses near-field communication to connect the physical and logical worlds, as well as remote sensor organisers for data and vaccination security [29]. (Algorithm 1 shows the Pseudocode for blowfish, and algorithm 2 shows the Pseudocode for MD5.)

#### 3.4.2. MD5 Algorithm

A 512 bit block of information (each of which has sixteen 32 bit subblocks) makes up an MD5 message (Message-Digest algorithm). There are four connected 32 bit barriers to document honesty in MD5's 128 bit message processing.

### 3.5. Key Generation and Authentication

As soon as the patient register, a physician will be assigned. For initial password-based validation, physicians and patients use a secure password (MD5)-based technique. When a doctor logs into the system and views patient data, the system compares the doctor's palm/thumb picture scan reading to the recorded information. Access to the system will be granted after the doctor's ID is validated to ensure authenticity. The patient's palm/thumb is used to produce a biological key for security purposes.

### 3.6. Decryption

Decryption will begin only once authentication has been performed by extracting an authentication code or frame value from the previously created frame and completing the whole method for building an authentication code as specified. An encrypted version of the patient's data is stored in the MSWBAN Client, which comprises a collection of sensors and a control unit. Data consumers may check the cypher text's authenticity and decode the data if they have the decryption characteristics defined by the signature access structure when accessing data from the MSWBAN client [30].

#### 3.6.1. Performance Analysis

**Figure 5:**
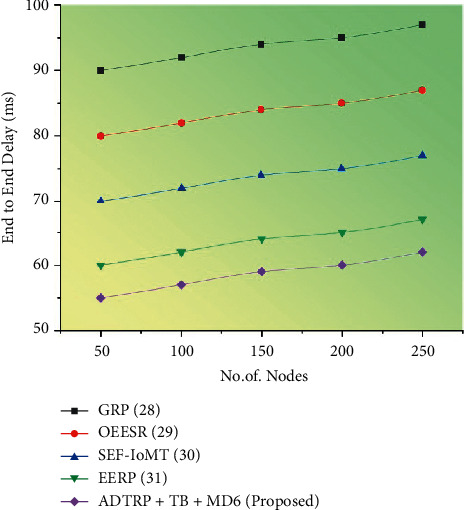
End-to-end delay results of the proposed methodology.

**Figure 6:**
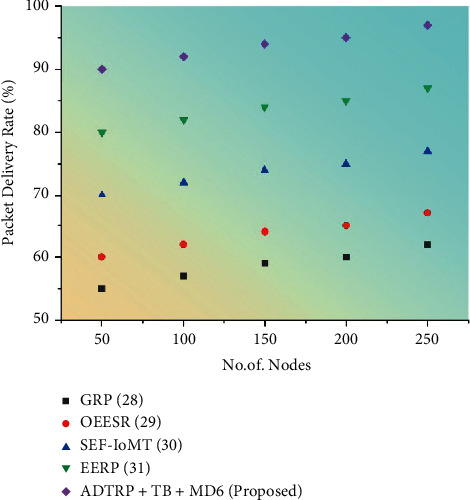
Packet delivery ratio results of the proposed methodology.

**Figure 7:**
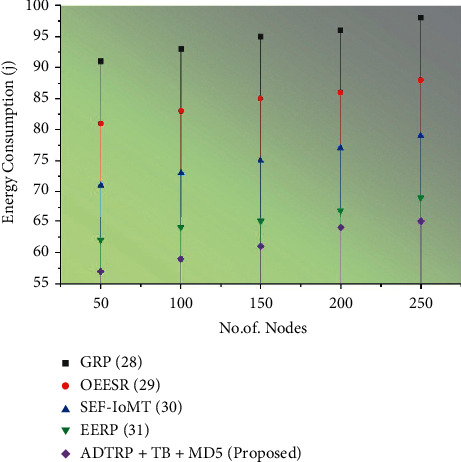
Energy consumption results of the proposed methodology.

**Figure 8:**
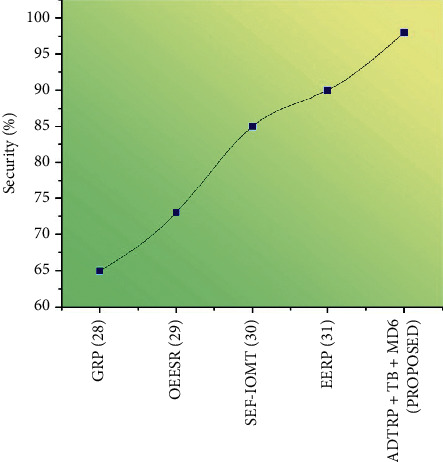
Comparison of security level for the existing and proposed methodology.

We conducted extensive simulation experiments using Castalia-3.2 simulator, built on the OMNeT++ platform, to verify our proposed work. Figures 5–8 shows the comparison of performance metrics, namely packet delivery ratio, security level, throughput, and end-to-end delay. The approaches include the Geographic Routing Protocol (GRP), Optimized Energy Efficient Secure Protocol (EESR), Secure and energy-efficient framework-Internet of Medical Things (SEF-IoMT), Energy Efficient Routing Protocol (EERP), and (ADTRP + TD-MD5) additive distance threshold routing with trouped blowfish MD5.  Table 2 shows the comparative analysis of metrics for existing and proposed methods, and Table 3 represents the simulation parameters.

End-to-end latency is determined by calculating the total amount of time needed for data transmission from the sender node to the destination node. Then, the delay in normal mode is determined using the following formula:

(16) $$\begin{array}{c} D=\frac{1}{n}\overset{n}{\underset{i=1}{\sum }}\left(Tr_{i}-Ts_{i}\right)\times 1000\left[ms\right]\text{,} \end{array}$$

where *D* is the average end-to-end delay, *i* is the packet identifier, *Tr*_*i*_ is the reception time, *Ts*_*i*_ is the send time, *n* is the number of packets successfully delivered.

Figure 5 represents the end-to-end delay results with proposed and existing approaches. Every millisecond a packet travels from the sender to the receiver is counted as an end-to-end latency measurement (mS). From the above figure, compared to the existing methods such as the dual sink approach using WBAN, novel framework for software-defined WBAN, fragmentation through MAC IEEE 802.15.4 to delay performance, efficient and reliable direct diffusion routing protocol in WBAN, and the proposed method of ADTRP + TB-MD5 has low end-to-end delay. The previous approaches like GRP has 35%, OEESR has 30%, SEF-IMOT has 25%, and EERP has 20% for the packet delivery ratio. The proposed ADTRP + TB-MDS has an 18% of packet delivery ratio.

The sum of the number of packets received to the number of packets issued is known as the packet delivery ratio. The following formula is used to determine the packet delivery ratio:

(17) $$\begin{array}{c} Packet\;delivery\;ratio=\frac{\sum total\;packets\;received\;by\;all\;destination\;nodes}{\sum total\;packets\;send\;by\;all\;source\;nodes}\times 1000\text{.} \end{array}$$

Packet delivery ratio results with proposed and existing approaches are shown in Figure 6. Packet delivery ratio measures the proportion of sending packets to received packets. From the above diagram, compared to the current methods such as GRP, OEESR and SEF-IoMT, and EERP, the proposed method of ADTRP + TB-MD5 has a high packet delivery ratio. The earlier methods, such as GRP, OEESR and SEF-IoMT, and EERP, had packet delivery ratios of 28%, 25%, 29%, and 33%, respectively. The suggested ADTRP + TB-MD5 has a packet delivery ratio of 40%. The current technique has a larger overall packet loss percentage during certain data transmission times in the sensor network than our recommended alternative.

Figure 7 represents the security level results with proposed and existing approaches. In the above diagram, compared to the current methods such as GRP, OEESR, SEF-IoMT, and EERP, the proposed method of ADTRP + TB-MD5 has high security.

Security is a wide concept that incorporates many different technologies, tools, and procedures. It is a collection of guidelines and settings intended to safeguard the reliability, accessibility, and integrity of computer networks and data.  Figure 8 shows the comparison of security levels for existing and proposed work. The security level of different encryption techniques is examined. GRP, OEESR, SEF-IoMT, and EERP, the proposed method of ADTRP + TB-MD5 has a high security level. The suggested approach of ADTRP + TB-MD5 has a high security level of 98%, whereas GRP achieves 65%, OEESR scores 73%, SEF-IoMT gets 85%, and EERP gets 90%.

The production rate of a specified process during a predetermined time period is known as throughput.

(18) $$\begin{array}{c} Throughput=\frac{Number\;of\;units\;produced}{Time\;period}\text{.} \end{array}$$

The most apparent goal of any efficient system is to increase throughput. However, remember that precision is more important than speed. When errors are made, productivity is reduced. The amount of data transmitted in a communication environment is referred to as throughput. It refers to the quantity of information or packets sent from the source node to the destination node. Throughput is calculated as the amount of traffic received minus traffic transmitted divided by the total number of data packets received.

**Figure 9:**
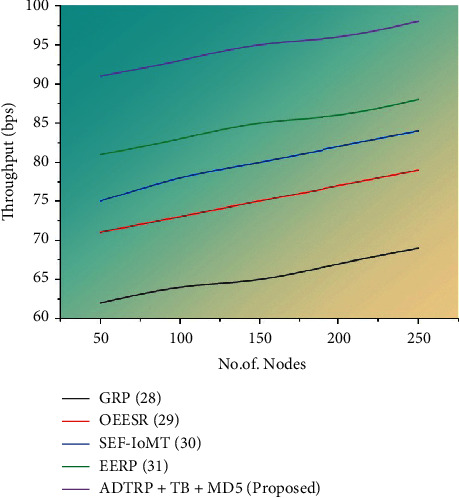
Throughput results of the proposed methodology.

Figure 9 represents the throughput results with proposed and existing approaches. Bits-per-second (BPS) is a unit of measurement for the quantity of data sent by a network system. The suggested approach of ADTRP + TB-MD5 has a high throughput of 98 bps when compared to the current methods at the 250th node, where GRP attains 69 bps, OEESR attains 79 bps, SEF-IoMT attains 84 bps, and EERP attains 88 bps.

**Figure 10:**
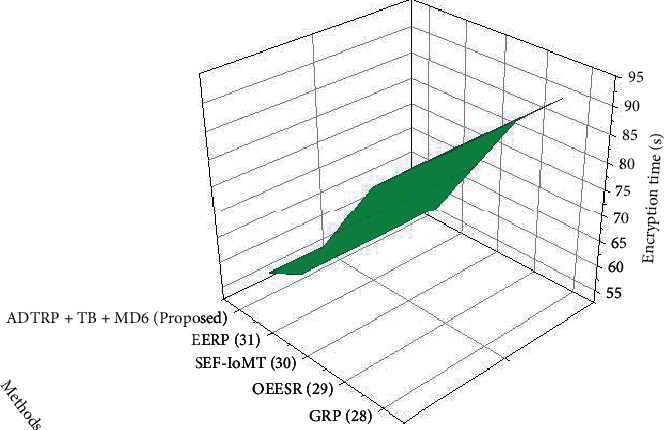
Encryption time results of the proposed methodology.

**Figure 11:**
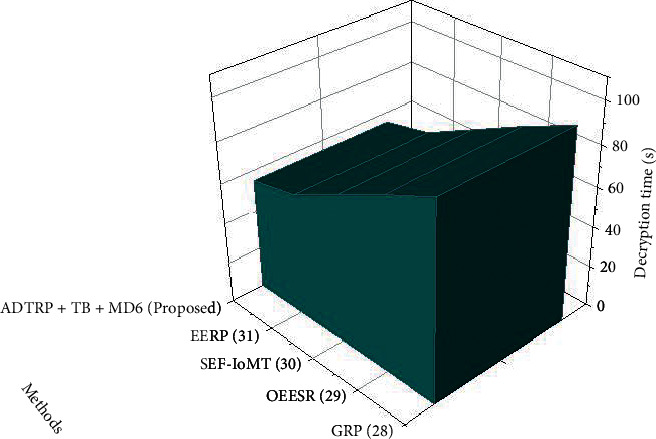
Decryption time results of the proposed methodology.

The encryption is the amount of time required to transform plaintext into ciphertext. In contrast, decryption time restores the plaintext from the received ciphertext. Decryption, on the other hand, recovers the plaintext from the received ciphertext. The speed of every algorithm is inversely related to the quantity of data it must process. For the encryption and decryption execution time, the suggested method and existing approach were compared. Figures 10 and 11 illustrate the outcomes. The figures show that the suggested technique requires less calculation time for encryption and decryption.

The overall comparison of proposed and existing methods shows that the proposed methods are high in security levels, packet delivery, and throughput and low in end-to-end delay.

## 4. Discussion

Figures 5–8 compare throughput, end-to-end delay, packet delivery ratio, the security level for the existing method, and proposed plans. The current approaches are the dual sink approach using WBAN, a novel framework for software-defined WBAN, fragmentation through MAC IEEE 802.15.4 to delay performance, efficient, and reliable direct diffusion routing protocol in WBAN, and the proposed method is additive distance threshold routing protocols (ADTRP + TB-MD5). The proposed method, ADTRP + TB-MD5 has an end-to-end delay of 63 ms, and the existing techniques GRP has 82 ms, OEESR has 7 ms, SEF-IoMT has 72 ms, EERP has 81 ms. So, compared to existing methods, the proposed plans perform better in terms of end-to-end delay. The proposed method, ADTRP + TB-MD5, has a packet delivery ratio of 95% and the existing techniques, GRP, has 90%, OEESR has 93%, SEF-IoMT has 92%, EERP has 81%. So, compared to existing methods, the proposed technique performs better in terms of packet delivery ratio. The security level of the proposed method ADTRP + TB-MD5 is 95.7%, and in the existing process, GRP is 81%, OEESR is 88%, SEF-IoMT is 87%, and EERP is 80%. Hence, compared to existing methods, the proposed techniques perform better in terms of security level. In terms of throughput delay, the proposed method, ADTRP + TB-MD5, has 9120 Mbps, and the existing plans, and GRP has 8460 Mbps, OEESR has 8830 Mbps, SEF-IoMT has 8786 Mbps, and EERP has 8086%. So, compared to existing approaches, the proposed method is better in terms of throughput. The overall comparison of all parameters shows that the proposed method performs better.

## 5. Conclusion

The detection and prevention of COVID-19 are major concerns all over the world. As a result of this research, a model for an energy-efficient multisensor wireless body area network that is capable of monitoring COVID-19 patients has been developed. When a user reports symptoms to the cloud, the additive threshold routing protocol analyzes them to determine whether or not the user has COVID-19. If a user has been reported as infected with COVID-19, the MSWBAN will always keep a record of their infection status in the database. It is possible that in the future, new categorization algorithms will be utilised to improve the MSWBAN's accuracy as well as its long-term viability. Using parallel and distributed processing based on microservices achieved through GPU grids, the proposed health application would be able to analyse multiple data flows coming from various devices for the purposes of machine learning and pattern recognition. In addition to that, and as was mentioned earlier, it will utilise federated learning approaches in order to monitor and artificially reason about data streams coming from a large number of MSWABNs.

## Figures and Tables

**Algorithm 1:**
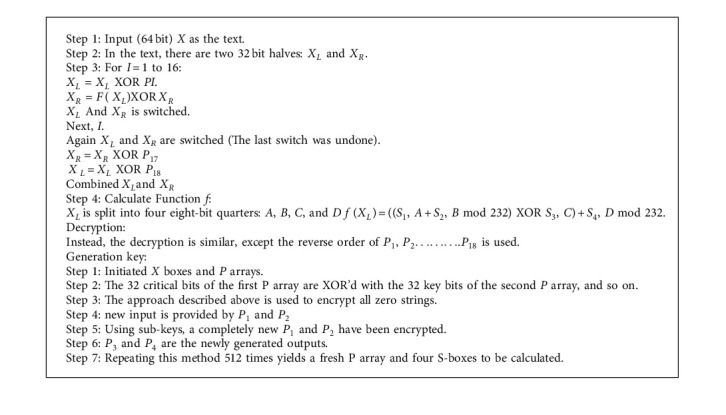
Pseudocode for blowfish.

**Algorithm 2:**
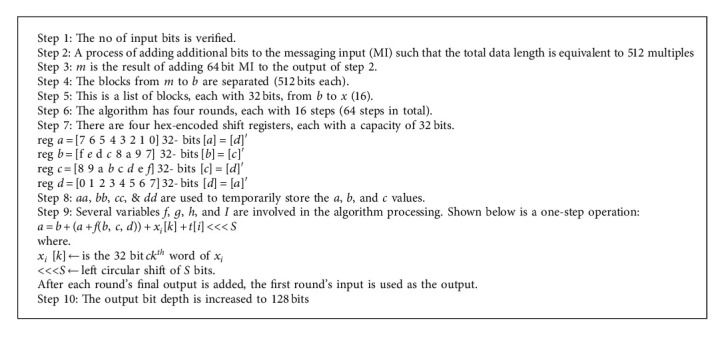
Pseudocode for MD5.

**Table 1:** Summary of related works.

Reference	Methods	Advantages	Drawbacks
Singla et al. [6]	Secure routing technique	The pricey secure data transfer is not required if no incident is found.	Bandwidth is wasted. It requires a high computational cost for encryption and needs more RAM.

Jabeen et al. [8]	Nanosensors	It gives a high surface area/volume ratio by increasing their sensitivity.	These sensors always adopt a similar fundamental process.

Majumder et al. [9]	Remote patient monitoring	Increasing communication options strengthen the patient-provider connection and raise customer loyalty and satisfaction.	It relies on technology, which some people may not be able to afford. Reliable Internet connections are required for RPM systems.

Kaur et al. [10]	Routing protocol	No route setup delay for connections over small distances. Reactive routing for farther-off destinations results in reduced routing overhead.	They depend on routable network protocols to function. Compared to other network devices, they are expensive.

Thomas and Suresh [18]	Hospital management	Every piece of data is accessible by approved login from anywhere in the globe. This form of communication has become considerably less expensive.	User interface and user experience (UI/UX design) are complex designs concerned with a data breach.

Rahman et al. [19]	WSN	Because it is scalable, any additional nodes or devices may be added at any moment.	Due to its limited speed architecture, it cannot be utilised for high-speed communication.

Paganelli et al. [25]	Multistage fuzzy rules	Fuzzy logic systems have a straightforward and reasonable structure. The fuzzy logic is typically applied in practical and business contexts.	In the large organization industry, it is used for dynamic, emotionally supporting networks and individual assessments.

**Table 2:** Comparative analysis of the proposed methodology.

S. no	Classification methods	End-to-end delay (ms)	Packet delivery rate (%)	Security level (%)	Throughput (bps)
(1)	GRP [31]	82	90	81	8460
(2)	OEESR [32]	7	93	88	8830
(3)	SEF-IoMT [33]	72	92	87	8786
(4)	EERP [33]	81	81	80	8086
(5)	ADTRP + TB-MD5 [proposed]	63	95	95.7	9120

**Table 3:** Simulation parameters.

S.no	Parameter	Value
(1)	No. of nodes	250
(2)	Time	270 s
(3)	Energy consumption	16.3 j
(4)	Transmission power	−15 dBm
(5)	No. of packets	250
(6)	Depth threshold	10 m
(7)	Min: and max: communication range	225 m, 255 m
(8)	Packet generation frequency	0.02 pkts/min
(9)	Transmission range	32 cm
(10)	Node displacement	0–5 m/s
(11)	Number of rounds taken for simulation	450 rounds
(12)	Number of sinks	1
(13)	Data processing rate	15,000 bits/s
(14)	Temperature threshold	45°C
(15)	SNR	16 dB

## Data Availability

The data used to support the findings of this study have been deposited at https://doi.org/10.1109/ISMS.2018.00031.

## Additional Points

The schematic representation of the suggested technique includes the process of analysing the fuzzy logic dependent cluster head selection, wireless body area network deployment, sending node, receiving node, encryption using the trouped blowfish MD5 algorithm, cloud database, key generation, and authentication, decryption, distant monitoring of COVID-19 patients using additive distance-threshold routing protocol in WBAN innovative system.
